# Management of anal cancer patients – a pattern of care analysis in German-speaking countries

**DOI:** 10.1186/s13014-020-01539-x

**Published:** 2020-05-25

**Authors:** Daniel Martin, Jens von der Grün, Claus Rödel, Emmanouil Fokas

**Affiliations:** 1 Department of Radiotherapy and Oncology, Goethe University Frankfurt, Theodor-Stern-Kai 7, 60590 Frankfurt, Germany; 2 Frankfurt Cancer Institute (FCI), Frankfurt, Germany; 3 German Cancer Research Center (DKFZ), Heidelberg, Germany; 4 German Cancer Consortium (DKTK), partner site: Frankfurt a. M, Heidelberg, Germany

**Keywords:** Anal cancer, Patterns of care, Survey, HIV, Imrt, Chemoradiotherapy

## Abstract

**Background:**

Radiotherapy dose and target volume prescriptions for anal squamous cell carcinoma (ASCC) vary considerably in daily practice and guidelines, including those from NCCN, UK, Australasian, and ESMO. We conducted a pattern-of-care survey to assess the patient management in German speaking countries.

**Methods:**

We developed an anonymous questionnaire comprising 18 questions on diagnosis and treatment of ASCC. The survey was sent to 361 DEGRO-associated institutions, including 41 university hospitals, 118 non-university institutions, and 202 private practices.

**Results:**

We received a total of 101 (28%) surveys, including 20 (19.8%) from university, 36 (35.6%) from non-university clinics, and 45 (44.6%) from private practices. A total of 28 (27.8%) institutions reported to treat more than 5 patients with early-stage ASCC and 42 (41.6%) institutions treat more than 5 patients with locoregionally-advanced ASCC per year.

Biopsy of suspicious inguinal nodes was advocated in only 12 (11.8%) centers. Screening for human immunodeficiency virus (HIV) is done in 28 (27.7%). Intensity modulated radiotherapy or similar techniques are used in 97%. The elective lymph node dose ranged from 30.6 Gy to 52.8 Gy, whereas 87% prescribed 50.4–55. 8 Gy (range: 30.6 to 59.4 Gy) to the involved lymph nodes. The dose to gross disease of cT1 or cT2 ASCC ranged from 50 to ≥60 Gy. For cT3 or cT4 tumors the target dose ranged from 54 Gy to more than 60 Gy, with 76 (75.2%) institutions prescribing 59.4 Gy. The preferred concurrent chemotherapy regimen was 5-FU/Mitomycin C, whereas 6 (6%) prescribed Capecitabine/Mitomycin C. HIV-positive patients are treated with full-dose CRT in 87 (86.1%) institutions. First assessment for clinical response is reported to be performed at 4–6 weeks after completion of CRT in 2 (2%) institutions, at 6–8 weeks in 20 (19.8%), and 79 (78%) institutions wait up to 5 months.

**Conclusions:**

We observed marked differences in radiotherapy doses and treatment technique in patients with ASCC, and also variable approaches for patients with HIV. These data underline the need for an consensus treatment guideline for ASCC.

## Introduction

The standard treatment in anal squamous cell carcinoma (ASCC) is 5-FU and mitomycin C (MMC)-based chemoradiotherapy (CRT) [1–3]. Since the introduction of CRT by Nigro et al. [4] standard treatment has remained largely unchanged, despite technological advances in RT that facilitate better sparing of normal tissues to reduce acute and late toxicities [5].

Several international guidelines covering the staging and treatment of ASCC are available (ESMO-ESSO-ESSO, UK, Australasian, NCCN) [6–9] that are characterized by variability in dose prescription to the primary tumor as well as the elective and involved lymph nodes. Dosing recommendations to the primary tumor range from 50.4 to 60 Gy - in some guidelines according to T stage. Classic shrinking field technique as is reported in the NCCN and Australasian guidelines proposes a dose to the elective nodal volume from 30.6 or 36 Gy (for the inguinal region), and for simultaneous integrated boost (SIB) techniques to the elective lymph nodes, a dose of 40 to 45 Gy in 28 to 30 fractions [8, 9].

Also, human immunodeficiency virus (HIV) positive patients – especially in men who have sex with men (MSM) – are at a significantly higher risk to develop ASCC [10], and these patients were excluded from all major randomized trials due to expected toxicities. Nevertheless, in the era of combined antiretroviral therapy several retrospective reviews indicate the feasibility of standard CRT with comparable outcomes [11–13].

As the incidence of ASCC is rising, especially due to the association with human papilloma virus infection (HPV) [14, 15], there is a need to harmonize treatment recommendations. To date, there are no S3-guidelines for the treatment of ASCC in German-speaking countries. As such, we conducted a pattern of care survey to gain insight into how patients with ASCC are treated in the clinical routine.

## Methods

### Survey

An anonymous questionnaire including 18 questions (16 single choice questions, 2 multiple choice questions) was created (Table 1), and an online survey was then generated using Google Forms® (Google, Mountain view, CA, USA). The link to the survey and the questionnaire PDF file for offline use was sent to 361 German Society of Radiation Oncology (DEGRO)-associated institutions, including German-speaking radiation oncology departments of 41 universities, 118 non-university institutions and 202 radiation oncology private practices in Germany, Austria and the German-speaking part of Switzerland. As the questions 8, 9 and 10 allowed individual answers regarding the prescribed radiation dose and all these individual answers covered dose ranges, we used the highest dose given for further analysis.

**Table 1:** Summary of the 18 questions used in our questionnaire

Question	
1. Are you employed at an academic hospital, community hospital or private practice?	Out-patient practice; Non-university Clinic; University Clinic
2. How many cases of ASCC do you treat per year according to stage?^a^	0; 1–5 or > 5 cases of early/advanced ASCC;
3. What imaging studies do you want in order to define the target volumes?^a^	MRI; CT; Endorectal US; PET-CT
4. Do you perform biopsy for suspicious inguinal lymph nodes?	Yes; No; Sometimes
5. Do you routinely screen patients with ASCC for HIV?	Yes; No; Only Male patients
6. What kind of RT technique do you use regularly for ASCC?	3D-RT; IMRT; Rotational IMRT; Tomotherapy
7. Do you use SIB techniques regularly?	Yes; No
8. What is the dose you prescribe for the elective lymph node areas?	30.6 Gy; 36 Gy; 45 Gy; 50.4 Gy; other
9. What is the dose you prescribe for the involved lymph node areas?	30.6 Gy; 36 Gy; 45 Gy; 50.4 Gy; 54–55.8 Gy; other
10. What is the dose you prescribe for T1/T2 tumors?	50–50.4 Gy; 54–55.8 Gy; 59.4 Gy; > 60 Gy; other
11. What is the dose you prescribe for T3/T4 tumors?	50–50.4 Gy; 54–55.8 Gy; 59.4 Gy; > 60 Gy
12. Do you use alternative boost techniques in the clinical routine?	No; Brachytherapy; Electron Boost
13. What is the regularly used chemotherapy regime in your department?	5-FU/MMC; Cap/MMC; 5-FU/Cis; Cap/Cis; Other
14. Do you treat HIV positive patients with standard dose CRT?	Yes; No, only with dose reduction; No
15. Do you prescribe adjuvant chemotherapy after primary CRT?	No; Sometimes; Yes regularly
16. Do you prescribe induction chemotherapy?	No; Sometimes; Yes regularly
17. Do you systematically evaluate QoL/PROM data during f/u?	Yes; No
18. At what timepoint do you assess the response status?	4–6 weeks; 6–8 weeks or up to 5 months after end of treatment

All questions are single choice unless described otherwise

Abbreviations: *HIV,* human immunodeficiency virus, *ASCC*, anal squamous cell carcinoma, *MRI* magnetic resonance imaging, *MMC* Mitomycin C, *Cap* Capecitabine, *Cis* Cisplatin, *QoL* Quality of Life, *PROM* Patient reported outcome measurements, *f/u* follow up;

^a^ multiple choice questions

### Statistical analysis

Frequency distributions of responses for each question were calculated. The answers were also analyzed by type of institution (university department vs. non-university institution vs out-patient practices) using Pearson’s chi-squared test. All statistical analysis was performed using the R software package (Version 3.6) [16].

## Results

We received 101 answers from 361 inquiries (28%), including 20 (19.8%) from university departments, 36 (35.6%) from non-university clinics and 45 (44.6%) from out-patient radiation oncology practices. A total of 28 (27.8%) institutions reported to treat more than 5 patients per year with early ASCC, defined as cT1-2N0M0, and 42 (41.6%) institutions treat more than 5 patients annually with locoregionally advanced ASCC (Table 2). Routine screening for infection with HIV for all patients is done in 28 (27.7%) institutions, and in additional 5 (4.9%) only male patients are screened. HIV positive patients are treated with standard CRT without dose reduction in 87 (86.1%) institutions, while 5 (4.9%) use a reduced dose of chemotherapy, while 5 (4.9%) do not prescribe concurrent chemotherapy at all.

**Table 2:** Staging related answers

	University (A) vs. Non-university Clinic (B) vs. out-patient practice (C)			***p***-Value
Total no of answers: 101	A	B	C	
*Number of cases per year; early ASCC*	N(%)	N(%)	N(%)	
< 5 with early ASCC	10 (45)	24 (67)	31 (69)	
≥ 5 with early ASCC	9 (50)	10 (28)	9 (20)	0.15
Not answered^b^	1 (5)	2 (5)	5 (11)	
*Number of cases per year; advanced ASCC*				
< 5 with advanced ASCC	6 (30)	19 (53)	31 (69)	
≥ 5 with advanced ASCC	13 (65)	17 (47)	12 (27)	**0.02**
Not answered^b^	1 (5)	0 (0)	2 (4)	
*Staging (multiple selection possible)*				
CT pelvis	11 (55)	25 (69)	34 (76)	
MRI pelvis	19 (95)	31 (86)	36 (80)	
Endorectal US	7 (35)	22 (61)	27 (60)	
PET-CT	7 (35)	8 (22)	3 (7)	Not done^a^
*Inguinal biopsy of suspect nodes*				
Always	2 (10)	5 (14)	5 (11)	
Sometimes	10 (50)	13 (36)	22 (49)	
Never	8 (40)	18 (50)	18 (40)	0.80
*Routine HIV screening*				
Yes	11 (55)	10 (28)	7 (16)	
No	8 (40)	23 (64)	37 (82)	
Only male patients	1 (5)	3 (8)	1 (2)	**0.01**

Abbreviations: *HIV* human immunodeficiency virus, *ASCC* anal squamous cell carcinoma

^a^ As multiple selections were possible in this answer we did not conduct a chi square test

^b^ Questionnaires without valid answers to this question were not included into the chi square analysis

Mandatory imaging for RT treatment planning includes magnetic resonance imaging (MRI) of the pelvis in 86 (85%) institutions, 70 (69,3%) use a computed tomography (CT) of the pelvis, and 56 (55.4%) use endorectal ultrasound studies in addition to CT/MRI. Only 18 (17.8%) institutions apply a fluorodesoxyglucose-PET/CT for planning, 12 (11.8%) routinely perform a biopsy of suspicious inguinal lymph nodes, 44 (43.5%) never conduct a biopsy, whereas a biopsy is occasionally performed in 45 (44.5%) centers (Table 2).

We also evaluated the answers with regard to institution (university vs. non-university clinic vs. out-patient practice, Table 2). University hospitals treated significantly more patients with advanced ASCC, especially compared to out-patient practice (13, 65% vs. 17, 47% vs. 12, 27% for university vs. non-university clinic vs. out-patient practice, respectively; *p* = 0.02). Routine HIV-screening is done significantly more often in university clinics (11, 55% vs. 10, 28% vs. 7, 16% for university vs. non-university clinic vs. out-patient practice, respectively; *p* = 0.01).

Most institutions use intensity modulated radiotherapy (IMRT) (24%) or rotational techniques (e.g. VMAT, RapidArc; tomotherapy; 73%) (Table 3). A large variability was observed regarding the prescribed radiation dose to clinical target volumes (Table 4). The dose to gross disease of cT1 or cT2 ASCC ranged from 50 to ≥60 Gy, with 83 (82.1%) using 54–59.4 Gy. For cT3 or cT4 tumors the target dose ranged from 54 Gy to more than 60 Gy, with 76 (75.2%) institutions prescribing 59.4 Gy. The doses to the elective lymph node CTV ranged from 30.6 Gy to 52.8 Gy. The dose range for the involved lymph node CTV was even greater, ranging from 30.6 to 59.4 Gy, but most institutions (88, 87.1%) prescribed doses from 50.4–55.8 Gy.

**Table 3:** Treatment related answers

	University (A) vs. Non-university Clinic (B) vs. out-patient practice (C)			***p***-Value
Total no of answers: 101	A	B	C	
*RT modality*	N(%)	N(%)	N(%)	
3D	0 (0)	1 (3)	2 (4)	
IMRT	4 (20)	8 (22)	12 (27)	
Rotational IMRT (e.g. VMAT; RapidArc)	14 (70)	26 (72)	31 (69)	
Tomotherapy	2 (10)	1 (3)	0 (0)	0.43
*Routine use of a SIB*				
Yes	6 (30)	15 (42)	19 (42)	
No	14 (70)	21 (58)	26 (58)	0.61
*Use of alternative boost techniques*				
Electrons	2 (10)	6 (17)	1 (2)	
Brachytherapy	4 (20)	9 (25)	2 (5)	
No	14 (70)	21 (58)	41 (93)	**< 0.01**
*Standard CTx regime*				
5-FU/MMC	19 (95)	35 (97)	41 (91)	
Capecitabine/MMC	1 (5)	1 (3)	4 (9)	0.5
*Adjuvant CTx*				
Yes; regularly	0 (0)	0 (0)	1 (2)	
Yes; sometimes	4 (20)	4 (11)	3 (7)	
No	16 (80)	32 (89)	41 (91)	0.45
*Induction CTX*				
Yes; sometimes	0 (0)	1 (3)	1 (2)	
No	20 (100)	35 (97)	44 (98)	0.77
*Standard CTx for HIV+ patients?*				
Yes	18 (90)	31 (88)	38 (91)	
w/ dose reduction	2 (10)	2 (6)	1 (2)	
No	0 (0)	2 (6)	3 (7)	0.57
*Timepoint of response assessment*				
4–6 weeks after end of treatment	0 (0)	1 (3)	1 (2)	
6–8 weeks after end of treatment	6 (30)	4 (11)	10 (22)	
Up to 5 months after end of treatment	14 (70)	31 (86)	34 (76)	0.46
*Evaluation of QoL/PROM*				
Yes	4 (20)	13 (37)	10 (23)	
No	16 (80)	22 (63)	34 (77)	0.26

Abbreviations: *HIV* human immunodeficiency virus, *ASCC* anal squamous cell carcinoma

^a^ As multiple selections were possible in this answer we did not conduct a chi square test

^b^ Questionnaires without valid answers to this question were not included into the chi square analysis

**Table 4:** Radiotherapy Doses

	University (A) vs. Non-university Clinic (B) vs. out-patient practice (C)			***p***-Value
Total no of answers: 101	A	B	C	
*Dose to GTV T1/T2 Tumors*				
50–50.4 Gy	4 (20)	7 (19)	4 (9)	
54–55.8 Gy	10 (50)	19 (53)	29 (66)	
59.4 Gy	5 (25)	10 (28)	10 (23)	
> 60 Gy	1 (5)	0 (0)	1 (2)	0.60
*Dose to GTV T3/T4 tumors*				
54–55.8 Gy	6 (30)	2 (6)	5 (11)	
59.4 Gy	11 (55)	30 (83)	35 (78)	
> 60 Gy	3 (15)	4 (11)	5 (11)	0.1
*Dose to elective lymph nodes*				
30.6 Gy	0 (0)	2 (6)	1 (2)	
36 Gy	4 (20)	2 (6)	9 (20)	
45 Gy	13 (65)	22 (61)	29 (64)	
50.4 Gy	3 (15)	10 (27)	6 (13)	0.31
*Dose to involved lymph nodes*				
36 Gy	1 (5)	0 (0)	0 (0)	
45 Gy	1 (5)	1 (3)	3 (7)	
50.4 Gy	7 (35)	7 (19)	18 (40)	
54–55.8 Gy	9 (45)	25 (69)	22 (49)	
59.4 Gy	2 (10)	3 (8)	2 (4)	0.32

A simultaneously integrated boost (SIB) technique is used in 40 (39.6%) institutions, while a brachytherapy boost is used as alternative to a percutaneous boost in 15 (14.8%); 9 (8.9%) institutions use electrons for a percutaneous boost. Regarding the use of alternative boost techniques, we found that techniques like brachytherapy or electron boosts are mostly used in non-university clinics and the least used in out-patient practice (6, 30% vs. 15, 43% vs. 3, 7% for university vs. non-university clinic vs. out-patient practice, respectively; *p* <  0.01).

The standard 5-FU/Mitomycin C-based CRT is performed in 95 (94%) centers, whereas 6 (6%) use Capecitabine/Mitomycin C as concomitant chemotherapy. Consolidation chemotherapy is prescribed routinely in only 1 institution, whereas 11 (10.9%) institutions occasionally prescribe consolidation chemotherapy; 2 (2%) departments also prescribe induction chemotherapy on a case by case basis. There were no significant differences regarding radiotherapy technique and dose, or use of concurrent chemotherapy, between institutions.

Regarding the timepoint for assessing clinical response, 2 (2%) institutions use 4–6 weeks, 20 (19.8%) use 6–8 weeks, and 79 (78.2%) wait up to 5 months after end of treatment for final response assessment (Table 3). Routine evaluation of patient’s quality of life (QoL) and patient-reported outcome (PROMs) is performed in 27 (26.7%) institutions (Table 3).

## Discussion

This is, to the best of our knowledge, the first pattern of care survey for ASCC in German-speaking radiation oncology institutions. With regard to pretreatment staging modalities, MRI of the pelvis is mandatory in most institutions, whereas a FDG-PET/CT is done in a minority, likely due to the lack of reimbursement by health insurances in Germany. Nevertheless, previous data describe FDG-PET/CT as a helpful imaging modality as it can lead to up- and downstaging in 28% of patients [8, 17, 18]. Additionally, FDG-PET/CT can be of use in the detailed contouring of the target volume [19], which is very important in the era of IMRT as a geographical miss can have a large impact on prognosis [20]. A large discrepancy between institutions was observed for diagnostic biopsy of suspicious inguinal lymph nodes. The current NCCN guideline recommends considering fine needle aspiration biopsy for suspicious inguinal nodes [8] and the ESMO-ESSO-ESTRO guidelines reports that it is usually only performed for patients with palpable lymph nodes or those enlarged > 10 mm in imaging studies [6].

Routine screening for HIV is advocated by the current NCCN guidelines because of the higher incidence of ASCC in HIV-positive patients and the non-negligible number of HIV-infected patients in the United states that are not aware of their infection status [8]. The Centers for Disease Control and Prevention (CDC) recommend HIV screening for all patients in health care settings [21], whereas the European guidelines consider HIV screening as optional but recommended [6]. In our survey, routine screening is only offered in 27%, which certainly shows that improvement in this area is needed in order to improve patients care. Interestingly, 5% of institutions offer HIV testing routinely only to male patients. The reasoning behind must be the higher probability of male patients being HIV positive, nevertheless expanding this to all patients is recommended. Treating HIV-positive patients with standard CRT was thought to be associated with more toxicities due to the immune compromised state of these patients, nevertheless several retrospective series reported comparable toxicities for patients that are under combined antiretroviral therapy [11, 13]. The NCCN and ESMO-ESSO-ESTRO guidelines support standard CRT for HIV-positive patients that are under combined antiretroviral therapy, despite missing evidence from randomized studies [6, 8].

As reflected in the different guidelines (ESMO-ESSO-ESSO, UK, Australasian, NCCN) [6–9], we observed a rather large variability in the RT doses used for the primary tumor, as well as for involved and elective lymph nodes among the different German-speaking institutions. The ongoing PLATO umbrella trial, containing the ACT3, ACT4 and ACT5 trial, currently assesses the efficacy and toxicity of risk-adapted RT in ASCC [22]. ACT3 is a non-randomized phase II trial for patients with T1N0 tumors after local resection. Depending on resection margins, patients either receive no adjuvant therapy (margins > 1 mm) or receive adjuvant CRT with MMC and capecitabine and a total RT dose of 41.4 Gy. ACT4 is a randomized phase II trial in patients with T1/2 (up to 4 cm) N0 tumors. Patients in the standard arm receive MMC/capecitabine-CRT with 50.4 Gy to the tumor and 40 Gy to the elective nodal region, which is reduced to 41.4 Gy and 34.5 Gy, respectively, in the experimental arm. ACT5 recruits patients with advanced ASCC (T2N1–3 or T3/4Nany). There are three arms in this trial, the standard arm consists of MMC/capecitabine-CRT up to 53.2 Gy, while patients in both experimental arms are treated to a dose of either 58.8 or 61.6 Gy in 28 fractions. These studies will provide important data on the optimal risk-adapted RT dose for different risk-groups of ASCC.

Using IMRT instead of conventional 3D-RT has been adopted in the vast majority of German-speaking institutions (97%), which is in line with the recommendations of the NCCN and ESMO-ESSO-ESTRO guidelines [6, 8]. The use of simultaneously integrated boost (SIB) techniques to the primary tumor was common (40.5%) among our survey. Although there is no randomized evidence that supports the use of SIB in ASCC, the RTOG 0529 trial – a single armed phase 2 study that used a dose painting technique - showed a trend towards a more favorable toxicity profile [23]. The current British guidelines also recommend a dose painting approach with 53.2 Gy in 28 fractions (1.9 Gy/fx) for the gross tumor volume of locoregionally advanced ASCC [7]. As it was not included into the survey we cannot comment on the contouring guidelines that are used in the institutions. Alternative boost techniques like brachytherapy only play a role in a minority of institutions. Dose escalation was prominently tested in the ACCORD 03 phase 3 trial, which showed no benefit for escalation beyond 60 Gy [3]. However, a recently published pooled analysis of ACCORD 03 and the KANAL phase 2 trial revealed that total doses of beyond 60 Gy might be associated with a better colostomy free survival [24]. Note that approximately half of the patients in this analysis were treated with brachytherapy. Additionally, a retrospective review suggests a role for brachytherapy in patients with non-complete response after completion of CRT [25]. The use of consolidation chemotherapy in our survey is very low – in line with the current evidence that suggests no benefit for induction- or consolidation chemotherapy [1, 2].

Recent results of the ACT II trial showed that the best time-point for assessment of treatment response is 26 weeks upon initiation of CRT, i.e. approximately 5 months upon CRT completion [26]. In our survey, the majority of departments (79, 78.2%) has adopted this longer waiting period. Due to ambiguous wording of the question the interpretation of the answers is difficult. Our intention was to assess the timepoint when the decision for response versus persisting disease is made regardless of diagnostics used.

There are some limitations to our study. The low response rate could lead to a biased analysis. Possible reasons for this could have been a) outdated contact information b) tightly scheduled routine work leading to a lack of time and c) lack of interest in the topic. The wording of question 18 was suboptimal and make analysis difficult. At last, we have no information on the used contouring guidelines.

## Conclusion

In conclusion, we observed several differences in radiotherapy doses and treatment techniques in ASCC, and also different management for HIV-positive patients among German-speaking radiation oncology institutions. These data further underline the need for a consensus treatment guideline for ASCC, which is currently prepared as S3 guideline in Germany and expected to be published in 2020.

## Data Availability

The datasets used and/or analysed during the current study are available from the corresponding author on reasonable request.

## Abbreviations

ASCC: Anal squamous cell carcinoma
CDC: Centers for disease control and prevention;
CRT: Chemoradiotherapy
Gy: Gray
HIV: Human immunodeficiency virus
IMRT: Intensity modulated radiotherapy
MMC: Mitomycin C
MSM: Men who have sex with men
SIB: Simultaneously integrated boost;
